# The response to adenosine tells all: What is the mechanism?

**DOI:** 10.1002/joa3.13154

**Published:** 2024-10-04

**Authors:** Shu Hirata, Koichi Nagashima, Yuji Wakamatsu, Ryuta Watanabe, Yasuo Okumura

**Affiliations:** ^1^ Division of Cardiology, Department of Medicine Nihon University School of Medicine Tokyo Japan

**Keywords:** accessory pathway, antidromic reciprocating tachycardia, Mahaim pathway, wide QRS tachycardia

## Abstract

The surface electrocardiograms during a wide QRS complex tachycardia and during the injection of 10 mg of adenosine triphosphate. What is the mechanism of this wide QRS tachycardia?

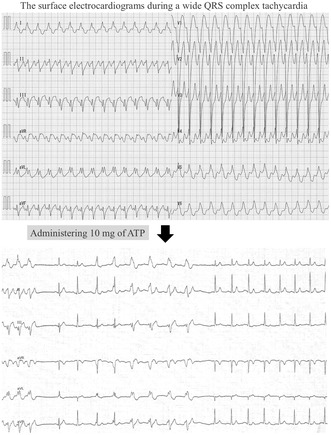

A 19‐year‐old man was referred to our hospital for the ablation of a wide QRS complex tachycardia (Figure 1A). The tachycardia was terminated by administering 10 mg of adenosine triphosphate (ATP) (Figure 1B). The electrocardiogram during sinus rhythm is also shown (Figure 1C). What is the mechanism of this wide QRS tachycardia?

**FIGURE 1:**
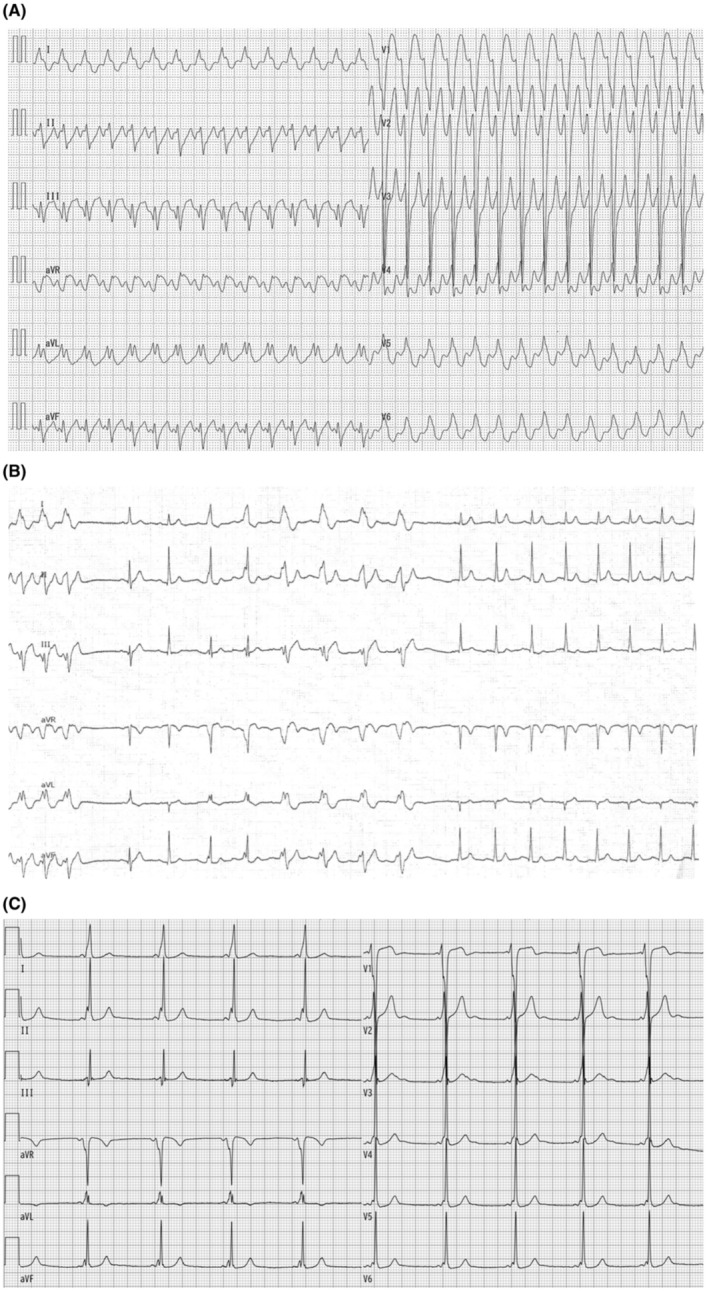
The surface electrocardiograms during (A) the tachycardia, (B) during injection of 10 mg of adenosine triphosphate, and (C) sinus rhythm.

The differential diagnoses of wide QRS tachycardias include ventricular tachycardia (VT), antidromic reciprocating tachycardia (ART) via an accessory pathway (AP), and supraventricular tachycardia (SVT)^1^ with aberrant conduction or a bystander AP. The key observations following ATP injection are as follows: ATP terminated the tachycardia with atrioventricular (AV) block, confirmed by the presence of a P wave detectable in the aVR lead. After termination, a narrow QRS beat was observed, followed by a gradual widening of the QRS complex along with PR interval prolongation, suggesting maximal pre‐excitation identical to the QRS morphology seen during tachycardia. This finding made VT less likely and indicated the presence of an AP. Additionally, these observations suggested that ATP had not yet blocked both anterograde and retrograde conduction through the AV node (AVN) but instead had blocked the AP conduction, resulting in the termination of the tachycardia. Therefore, the most likely diagnosis is ART with an ATP‐sensitive AP, rather than SVTs with aberrant conduction. Other ATP‐sensitive SVTs, such as atrioventricular nodal re‐entrant tachycardia or ATP‐sensitive atrial tachycardia with a bystander AP, were ruled out because the tachycardia was terminated by AV block, despite no effect on AVN conduction. The ATP‐sensitive AP was likely a classical Mahaim pathway, which includes nodoventricular and nodofascicular pathways, or a so‐called Mahaim pathway, including atriofascicular and atrioventricular pathways. Upon further review of the electrocardiogram during ATP injection, the recovery to normal sinus rhythm was divided into two periods: The first from tachycardia termination to the first two beats with narrow QRS, and the second from subsequent transition beats to maximal preexcitation. The first period indicated ATP's effect on the AP, while the second period indicated its effect on the AV node. This distinct effect suggested that the AP and AVN were independent in both proximal and distal portions, excluding nodoventricular and nodofascicular pathways, and favoring the atriofascicular or atrioventricular pathway.

Multielectrode catheters were positioned in the high right atrium, His bundle region, coronary sinus (CS), tricuspid annulus (TA), and right ventricular (RV) apex. During the electrophysiology study, a decrease in the coupling interval of the atrial extrastimulus revealed the stimulus‐QRS interval prolongation and a gradual change in QRS morphology, accompanied by a shortening of the His‐ventricular interval (Figure 2).^2^ This tachycardia was reset by a scanned atrial extrastimulus during septal atrial refractoriness (Figure 3). The ventricular electrogram in the His catheter occurred 70 ms (>33 ms) after the QRS onset, and the His electrogram was further delayed compared to the ventricular electrogram.^3^ Given these observations, ART via an atrioventricular Mahaim pathway was diagnosed. The pathway was eliminated by the radiofrequency application at the 9 o'clock position of the tricuspid annulus where the discrete Mahaim potential was not observed.

**FIGURE 2:**
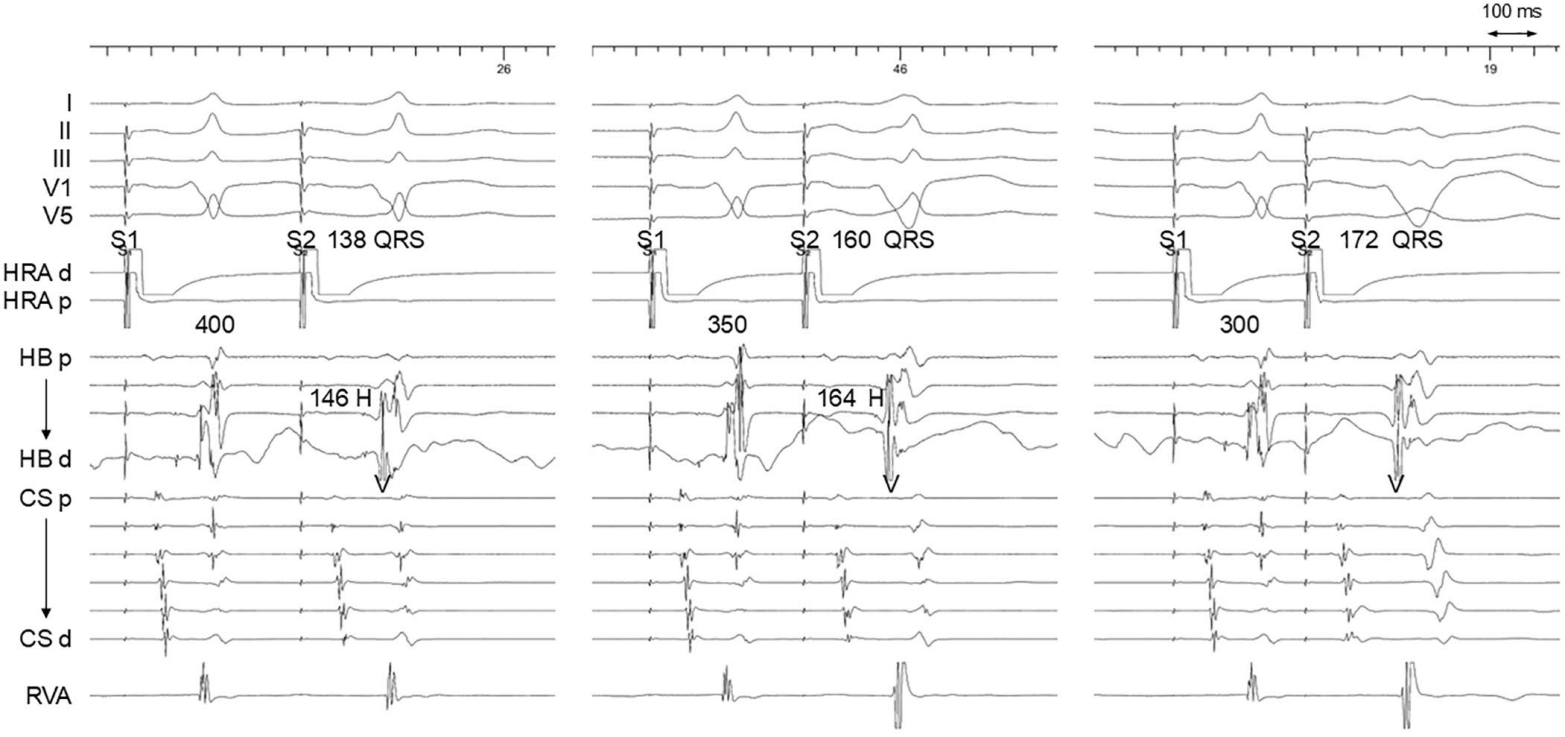
Intracardiac electrograms after atrial extrastimulus (S1–S1 = 600 ms, S1–S2 = 400 ms, 350 ms, and 300 ms). CS, coronary sinus; H, His; HB, His bundle; HRA, high right atrium; RVA, right ventricular apex; S, stimulus; V, ventricle.

**FIGURE 3:**
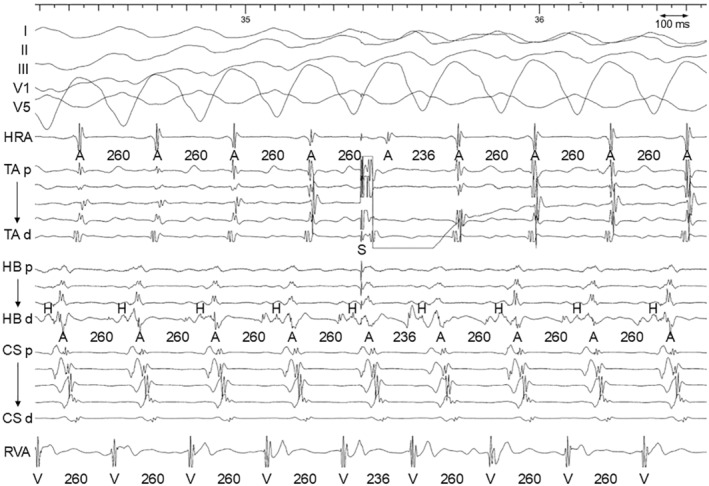
Intracardiac electrograms after a scanned atrial extrastimulus during atrial septum refractoriness. A, atrium; CS, coronary sinus; H, His; HB, His bundle; HRA, high right atrium; RVA, right ventricular apex; S, stimulus; TA, tricuspid annulus; V, ventricle.

The minimal preexcitation during sinus rhythm, despite the preexcited tachycardia, is the initial step in considering the Mahaim pathway. Continuous recording of electrograms during ATP injection for wide QRS tachycardia provides valuable clinical insights into further characterizing the AP.

## CONFLICT OF INTEREST STATEMENT

Authors declare no conflict of interests for this article.

## PATIENT CONSENT STATEMENT

The patient has provided consent for publication.

## PERMISSION TO REPRODUCE MATERIAL FROM OTHER SOURCES

Granted.

## STATEMENTS RELATING TO OUR ETHICS AND INTEGRITY POLICIES

The research related to human use complied with all relevant national regulations and institutional policies and is in accordance with the tenets of the Helsinki Declaration.
